# Genomic variation during culture adaptation of genetically complex *Plasmodium falciparum* clinical isolates

**DOI:** 10.1099/mgen.0.001009

**Published:** 2023-05-19

**Authors:** Antoine Claessens, Lindsay B. Stewart, Eleanor Drury, Ambroise D. Ahouidi, Alfred Amambua-Ngwa, Mahamadou Diakite, Dominic P. Kwiatkowski, Gordon A. Awandare, David J. Conway

**Affiliations:** ^1^​ LPHI, MIVEGEC, INSERM, CNRS, IRD, University of Montpellier, France; ^2^​ Department of Infection Biology, London School of Hygiene and Tropical Medicine, Keppel St, London, WC1E 7HT, UK; ^3^​ MRC Unit The Gambia at London School of Hygiene and Tropical Medicine, Banjul, The Gambia; ^4^​ Wellcome Sanger Institute, Cambridge, CB10 1SA, UK; ^5^​ Le Dantec Hospital, Université Cheikh Anta Diop, Dakar, Senegal; ^6^​ Malaria Research and Training Center, University of Bamako, Bamako, Mali; ^7^​ West African Centre for Cell Biology of Infectious Pathogens, Department of Biochemistry, Cell and Molecular Biology, University of Ghana, Legon, Ghana

**Keywords:** culture, loss-of-function mutants, mixed genotypes, genomic relatedness, adaptation

## Abstract

Experimental studies on the biology of malaria parasites have mostly been based on laboratory-adapted lines, but there is limited understanding of how these may differ from parasites in natural infections. Loss-of-function mutants have previously been shown to emerge during culture of some *Plasmodium falciparum* clinical isolates in analyses focusing on single-genotype infections. The present study included a broader array of isolates, mostly representing multiple-genotype infections, which are more typical in areas where malaria is highly endemic. Genome sequence data from multiple time points over several months of culture adaptation of 28 West African isolates were analysed, including previously available sequences along with new genome sequences from additional isolates and time points. Some genetically complex isolates eventually became fixed over time to single surviving genotypes in culture, whereas others retained diversity, although proportions of genotypes varied over time. Drug resistance allele frequencies did not show overall directional changes, suggesting that resistance-associated costs are not the main causes of fitness differences among parasites in culture. Loss-of-function mutants emerged during culture in several of the multiple-genotype isolates, affecting genes (including *AP2-HS*, *EPAC* and *SRPK1*) for which loss-of-function mutants were previously seen to emerge in single-genotype isolates. Parasite clones were derived by limiting dilution from six of the isolates, and sequencing identified *de novo* variants not detected in the bulk isolate sequences. Interestingly, several of these were nonsense mutants and frameshifts disrupting the coding sequence of *EPAC*, the gene with the largest number of independent nonsense mutants previously identified in laboratory-adapted lines. Analysis of genomic identity by descent to explore relatedness among clones revealed co-occurring non-identical sibling parasites, illustrative of the natural genetic structure within endemic populations.

## Data Summary

All data are fully provided in the Supplementary Material or through submission to public databases. The newly described parasite genome sequence data have been deposited in the European Nucleotide Archive. Accession numbers are all listed in Tables S1 (for clinical isolates at different time points in culture) and S3 (for parasite clones derived from clinical isolates) (available in the online version of this article).

**Impact Statement**

This is the largest study of genome sequence changes occurring in malaria parasites during continuous culture after isolation from clinical samples. Focusing on *Plasmodium falciparum*, the most important eukaryotic pathogen of humans, this study builds on earlier investigations by adding new clinical isolates as well as sequences from additional sample time points and clones from previous isolates, all of which were sampled from patients in West Africa, where the infection is endemic. This enabled more analysis of multiple-genotype isolates, to complement previous analyses that had mostly focused on single-genotype isolates. The results provide insights into processes of parasite adaptation to culture conditions, and will be useful in the design of prospective studies to identify mechanisms distinct from those occurring in natural populations.

## Introduction

Understanding the evolution and adaptation of eukaryotic pathogens requires special efforts, beyond those applied to understanding pathogens with smaller genomes or with higher mutation rates. Malaria parasites are highly adaptive to immunity and to chemotherapeutic or preventive interventions, and with haploid genomes of ~23 Mb and 14 well-characterized chromosomes they are more amenable than most eukaryotes to systematic study of evolution. The species of greatest medical importance is *Plasmodium falciparum*, one of only two malaria parasite species that have yet been successfully cultured continuously in the laboratory. Understanding parasite adaptation and the processes of evolution may be advanced by deeper analysis of this species, including sampling and cultivation of diverse natural isolates that have not previously been adapted to laboratory conditions.

Previous studies on parasite sequence evolution during culture have focused on laboratory-adapted lines [1, 2], or on clinical isolates that each had single genotypes prior to culture [3, 4]. Analyses of culture adaptation in 12 different single-genotype clinical isolates from 2 West African countries have shown that premature stop codon mutants emerged in approximately half of the isolates during several months of culture [3, 4]. Notably, two gene loci were identified as having independent mutant stop codons emerging in multiple different isolates, an *AP2* transcription factor gene on chromosome 13 (PF3D7_1342900) and the *EPAC* gene on chromosome 14 (locus PF3D7_1417400, encoding Rap guanine nucleotide exchange factor). Stop codon mutants in other genes, including *SRPK1*, have only been seen emerging in single isolates so far, although the small number of isolates examined does not preclude that these genes may also be repeatedly affected. The emergence of independent but convergent mutants indicates that loss of function in these genes is likely to be adaptive in culture. This is supported by loss-of-function mutants of the *AP2* gene having a phenotypic effect on growth at variable temperatures, which suggests heat shock regulation (thus designated as the *AP2-HS* gene) [5], and by the occurrence of multiple independent stop codons in the *EPAC* gene in other long-term culture adapted lines [3].

For detecting emerging mutants, clinical isolates containing single parasite genotypes had previously been focused on, as these were relatively straightforward to analyse [3, 4], although in endemic populations most *P. falciparum* clinical isolates have multiple genotypes co-circulating in the blood [6]. Initial analysis of these more complex isolates during the process of early culture adaptation has indicated that in most cases there is a gradual loss of genomic diversity [4], but analysis of genomic changes and emergence of new mutants in such isolates has not yet been performed.

Here, to support a broader analysis that would include multiple-genotype isolates, we generated new sequence data for combination with those previously obtained in a study of 24 Ghanaian clinical isolates sampled over several months of culture [4]. These new data comprise additional time point samples for 15 of the Ghanaian isolates, as well as 3 culture time points from each of 4 other West African clinical isolates that had not previously been described, enabling analysis of parasite genome sequences in 28 *P*. *falciparum* isolates over time in culture. Isolates with multiple genomes demonstrated a range of changes in composition during culture adaptation, not explained by previously known fitness costs of drug resistance alleles or by *de novo* loss-of-function mutations that emerged in a few isolates at relatively low frequencies. Parasites were also cloned from cultures of six of the isolates, and sequencing of a few of these clones for each isolate revealed additional mutations, as well as co-occurrence of non-identical sibling parasites, which is a feature of natural infections [7, 8].

## Methods

### Clinical *P. falciparum* isolates from malaria patients

Blood samples were collected from *P. falciparum* malaria cases presenting at government health facilities in Ghana, Guinea, Mali and Senegal, for analysis of parasites at multiple time points after introduction to continuous *in vitro* culture. Twenty-four of the clinical isolates were from patients at Navrongo in the Upper East Region of northern Ghana, as described in previous analyses of parasites at 3 culture time points [4], with new data for 15 of these isolates presented here on parasite sequences pre-culture and at later time points in culture. Four other isolates had not previously been described; two were from patients at Faranah in Guinea, one was from a patient at Nioro du Sahel in Mali and one was from a patient at Pikine in Senegal, and for each of these the parasite sequences at multiple time points in culture are presented here.

For each isolate, up to 5 ml of venous blood was collected into a heparinized vacutainer (BD Biosciences, CA, USA), and approximately half of the blood sample volume was cryopreserved in glycerolyte at −80 °C, while the remainder was processed to extract DNA from parasites for whole-genome sequencing. In samples from Ghana, leukocytes were removed by density gradient centrifugation and passage through Plasmodipur filters (EuroProxima, Netherlands), while in samples from Guinea, Senegal and Mali leukocytes were removed by passage through CF11 powder filtration columns, as previously described [9, 10]. All infections analysed here contained *P. falciparum* alone, as determined by Giemsa-stained thick-film slide microscopy, except for one Ghanaian sample (isolate 290) that also contained *P. malariae* that does not grow in continuous culture.

### Parasite culture

Cryopreserved patient blood samples were transferred by shipment on dry ice to the London School of Hygiene and Tropical Medicine, where culture was performed. Samples were thawed from glycerolyte cryopreservation and *P. falciparum* parasites were cultured continuously at 37 °C using standard methods [11] as follows (no isolates were pre-cultured before the thawing of cryopreserved blood in the laboratory on day 0 of the study). The average original volume of cells in glycerolyte in each thawed vial was approximately 1 ml, which yielded an erythrocyte pellet of at least 250 µl in all cases. Briefly, 12 % NaCl (0.5 times the original volume) was added dropwise to the sample while shaking the tube gently. This was left to stand for 5 min, and then 10 times the original volume of 1.6 % NaCl was added dropwise to the sample, while shaking the tube gently. After centrifugation for 5 min at 500 ***g***, the supernatant was removed and cells were resuspended in the same volume of RPMI 1640 medium containing 0.5 % Albumax II (Thermo Fisher Scientific, Paisley, UK). Cells were centrifuged again, supernatant was removed and the pellet was resuspended at 3 % haematocrit in RPMI 1640 medium supplemented with 0.5 % Albumax II under an atmosphere of 5 % O_2_, 5 % CO_2_ and 90 % N_2_ at 37 °C, with orbital shaking of flasks at 50 r.p.m. Replacement of the patients’ erythrocytes in the cultures was achieved by dilution with fresh erythrocytes from anonymous donors every few days, so that after a few weeks of culture parasites were growing virtually exclusively in erythrocytes from new donors. Clinical isolates were cultured in parallel in separate flasks at the same time, so that the donor erythrocyte sources were the same for different isolates, enabling comparisons without confounding from heterogeneous erythrocytes.

### Generating parasite clones by limiting dilution

Parasitized erythrocytes were diluted to 2 ml^−1^ mixed with uninfected erythrocytes at 1 % haemotocrit, and 250 µl was transferred to individual wells of a 96-well plate (a probability of 0.5 parasites per well). On each plate, 12 wells with uninfected erythrocytes at 1 % haematocrit (negative control) and 12 wells initially having 100 parasitized erythrocytes per well at 1 % haematocrit (positive control) were used to monitor the presence of parasites by microscopy. The plate was gassed in a culture chamber with 5 % CO_2_, 5 % O_2_ and 90 % N and incubated at 37 °C. The medium in each well was replaced after 24 h with fresh complete medium and subsequently at days 4, 7, 10 and 14. Fresh erythrocytes were added at day 4 and all wells were diluted fivefold at day 14. Then 50 µl of positive and negative control wells were removed on day 11 for PCR targeting the serine tRNA ligase gene locus (PF3D7_0717700). The erythrocytes were pelleted by centrifugation and the blood pellet added to the PCR reaction mixture in a final volume of 5 µl using the KAPA Blood PCR kit (peqlab), which does not require a separate DNA extraction, using the recommended cycling conditions in the kit. At day 11, negative control wells remained PCR-negative whilst all positive control wells were positive by PCR. At day 21, 50 µl was removed from each of the cloning wells and PCR was performed as described above. PCR-positive wells at this point were assumed to contain parasite clones and culture of each of these was scaled up into 5 ml volumes at 1 % haematocrit.

### Genome sequencing of parasites at different time points in culture

DNA extracted from parasites at each of the assayed culture time points and each clone was analysed by whole-genome Illumina short-read sequencing. Library preparation, sequencing and quality control were performed following internal protocols at the Wellcome Sanger Institute, similarly to previous sequence generation from clinical isolates from these populations [6, 9, 10]. Parasite genomic DNA was enriched by selective whole-genome amplification, which does not have a significant effect on the within-sample parasite sequence composition and diversity [12]. Genetic variants were called using a pipeline developed by the MalariaGEN consortium (ftp://ngs.sanger.ac.uk/production/malaria/Resource/28), with short reads mapped to the *P. falciparum* 3D7 reference genome sequence [13] version 3 using the BWA algorithm. Single-nucleotide polymorphisms (SNPs) and short insertions–deletions (indels) were called using GATK’s best practices. Variants with a VQSLOD score <0 or within the large subtelomeric gene families (*var*, *rif*, *stevor*, *clag*, *surfin*, *Pfmc-2tm* and *resa*) were excluded, to focus on reliable scoring variants in the core genome.

### Within-isolate genomic diversity, emergence of new mutants and analysis of relatedness among clones

Estimation of the level of parasite diversity within each isolate relative to overall diversity across all isolates was performed using the *F*_WS_ index [14, 15] (a within-isolate fixation index between 0 and 1.0 that is inverse to the level of diversity, such that a value of 1.0 indicates an isolate with a single genotype and lower values pertain to isolates that have mixed genotypes), as previously applied to analysis of some of the culture time points of the Ghanaian clinical isolates [4]. Here, the analysis is performed on all of the individual time point samples, with *F*_WS_ calculations based on SNPs having sequence read depths of at least 20 for a given sample (this is a stringent cut-off but overall similar trends are seen if the analysis is performed including SNPs with lower read-depth cut-offs of either 10 or 5). Analysis of new mutants followed methods similar to those previously used in analysis of clinical isolates with single-genotype infections [3, 4], except that isolates with mixed-genotype infections were analysed in this study. Allele frequencies of intragenic SNPs and frameshift-causing indels covered by read depths of at least 10 were plotted for each time point, to scan for the emergence of new mutant alleles. The quality of the mapped sequence reads for each of the cases of putative mutants was inspected visually using Savant software [16]. Although SNP calling proved straightforward in mixed-infection isolates, indel calling was not generally reliable due to the mixed signal, so indels in single-genotype isolates and clones were focused on. Finally, to estimate genetic relatedness between genomes of different parasite clones, identity by descent (IBD) was calculated using hmmIBD with default parameters [17].

## Results

### Whole-genome sequencing of *P. falciparum ex vivo* and after up to 7 months of culture adaptation

**Fig. 1.**
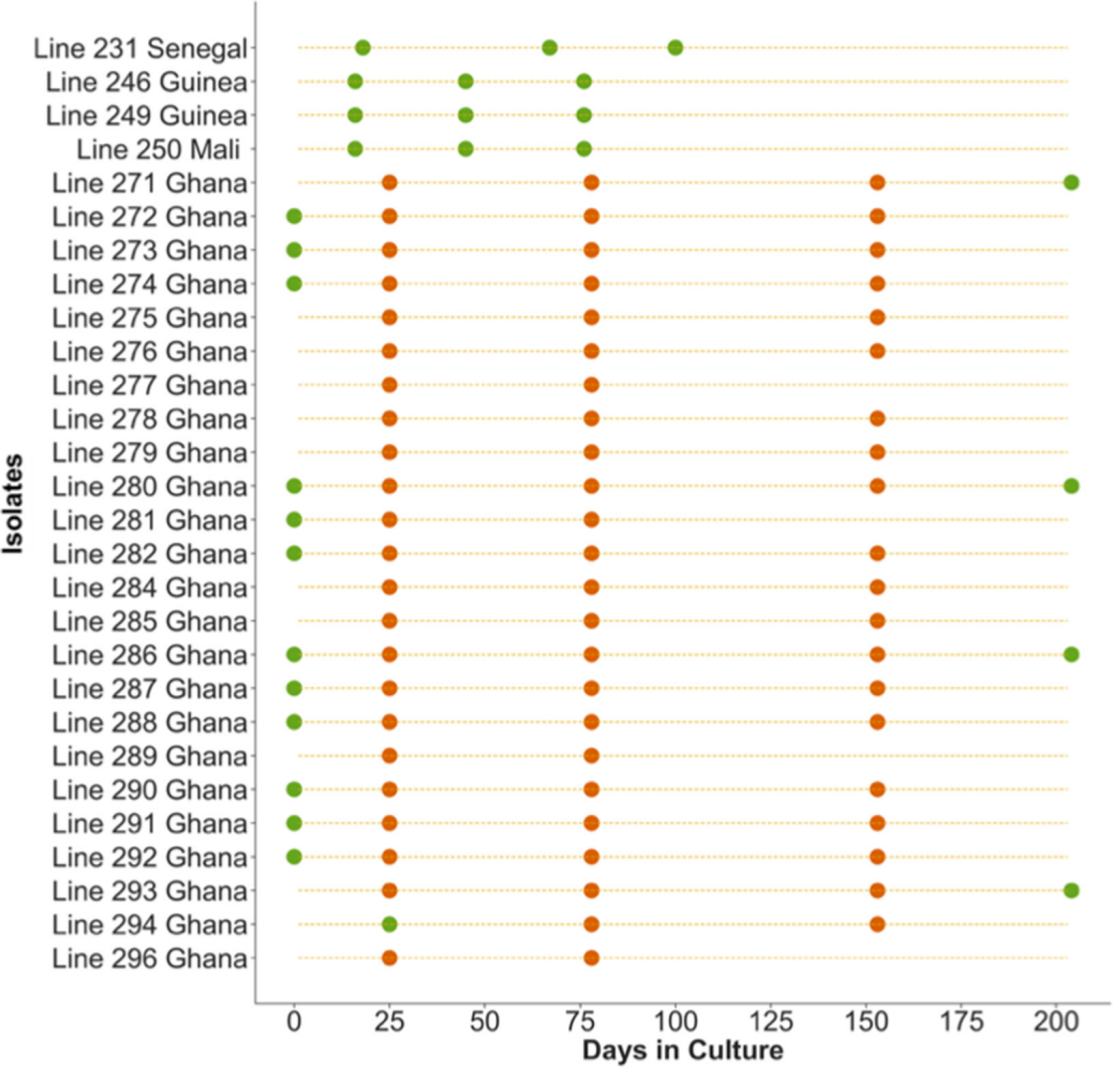
Scheme indicating sampling of genome sequences of *P. falciparum* clinical isolates at different time points during the process of culture adaptation. Circles indicate culture time points of 28 isolates sampled for whole-genome sequencing. Orange symbols indicate data previously obtained in a study of Ghanaian isolates, and green symbols indicate time points for which sequences were derived in the current study. The new data include additional time points for 15 of the Ghanaian isolates (including a pre-culture sample for 12 of these) as well as data for 4 isolates from other West African countries. Sequence accession numbers are given in Table S1.

Analysis of parasite genome sequence variation was performed on 28 *P*. *falciparum* clinical isolates from West Africa, grown in culture for periods of several months (range 76 to 204 days, median 153 days). The culture of each isolate was sampled for Illumina whole-genome sequencing on multiple occasions (a median of three and up to five time points) (Fig. 1). Overall, 96 high-quality bulk genome sequences from these uncloned parasite cultures are analysed here (Table S1), of which 67 sequences were previously generated in a study of Ghanaian isolates that analysed sequences of single-genotype isolates, but did not investigate novel variants within the multiple-genotype isolates [4]. The 29 new uncloned parasite genome sequences here comprised 17 additional time points for the Ghanaian isolates (including 12 at day 0 prior to culture) and 3 time points from each of 4 isolates from other West African countries (Fig. 1 and Table S1).

**Fig. 2.**
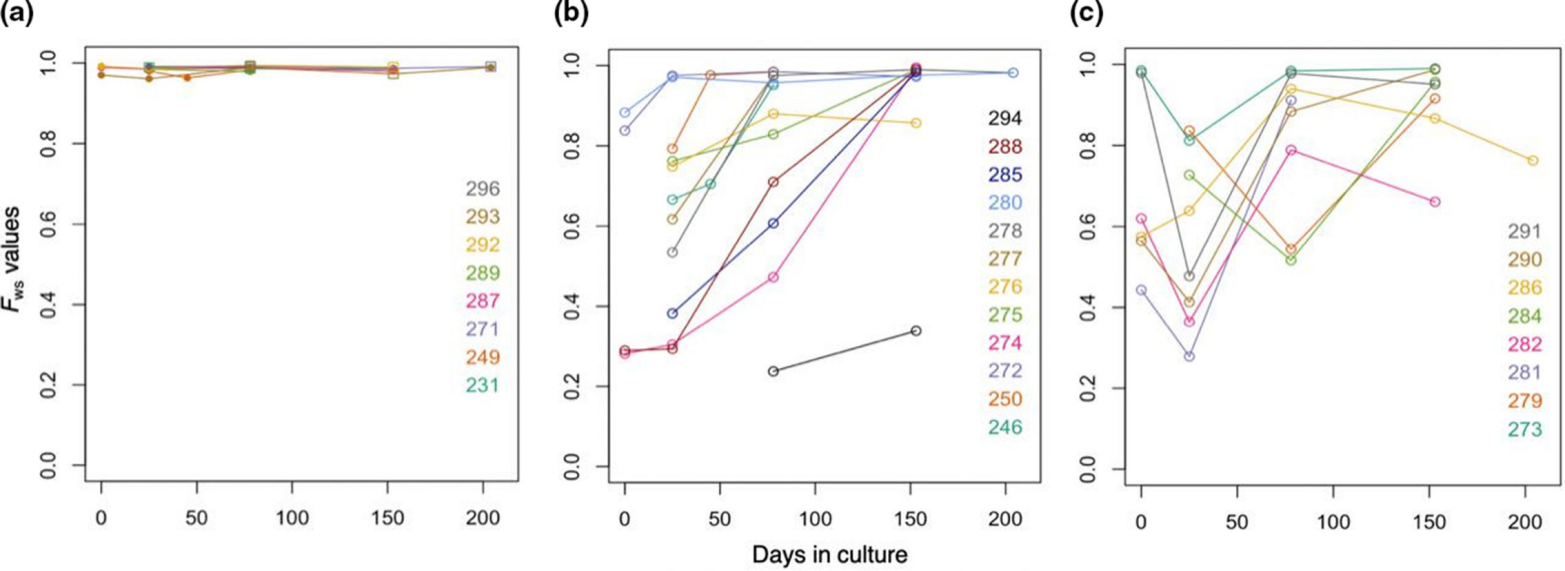
Different patterns of genomic diversity changes during culture of *P. falciparum* clinical isolates. Data for 28 different West African isolates are shown, with individual isolates being labelled by identification number and colour. For each time point, the within-isolate fixation index *F*_WS_ inversely indicates the genetic complexity, with values close to 1.0 indicating isolates having single genotypes and lower values indicating greater complexity. (**a**) Eight isolates show single genotypes throughout the culture adaptation period. (**b**) Twelve isolates each show progressive reduction of genetic diversity over time. (**c**) Eight isolates show more complex patterns, including a temporary increase in genetic diversity (*F*_WS_ index decreasing by a value of at least 0.1) occurring at some point during the culture adaptation period. The *F*_WS_ values for each individual sample time point for each isolate are given in Table S1. There is also a negative trend between *F*_WS_ values and varying levels of genome sequence read coverage, but this does not affect interpretation here as read coverage levels are not clustered by particular time points and do not differ overall across the data displayed in the three different panels of this figure (Table S1).

A summary measure of the genomic complexity of parasites within isolates was first obtained by calculating the *F*_WS_ fixation index, which ranges from 0 to 1 (an isolate with a value >0.95 having predominantly a single genome while lower values indicate more complex mixtures of different parasite genotypes). The overall trend was that within-isolate genomic diversity declined during culture adaption, as shown by the increasing *F*_WS_ index values over time (Fig. 2a, b), as previously noted for the isolates from Ghana [4]. However, in almost one-third of the isolates (8 out of 28), there were periods when the *F*_WS_ index decreased between successive time points, with declines of more than 0.1 in the values indicating temporary increases in diversity (Fig. 2c). As such patterns could reflect faster growing genotypes being sometimes initially rare within infections, or might reflect more complex processes, the profiles of allele frequency changes were next examined directly for each isolate.

### Genetic diversity within an isolate varies in different ways during culture adaptation

**Fig. 3.**
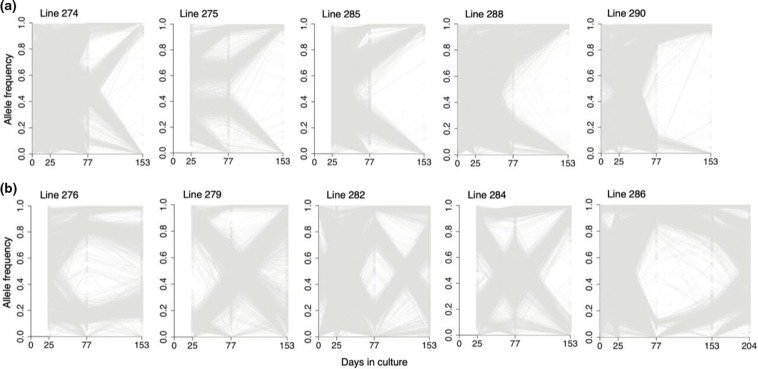
Genome-wide plots of SNP allele frequencies within *P. falciparum* multiple-genotype clinical isolates during culture adaptation. Allele frequency of each SNP within an isolate was estimated by the proportion of sequencing reads matching the reference (3D7 genome to which all sequences were mapped) or alternative allele for each nucleotide position. Grey lines indicate allele frequencies changing over time. Data for 10 of the isolates are shown. (**a**) Five isolates polyclonal at the first sequenced time point and monoclonal by day 153. (**b**) Five isolates polyclonal at the first sequenced time point, with variation in the proportion of each genome, that do not reach fixation by the end of the culture adaptation period. Plots for all isolates, including those not illustrated here, are shown in Fig. S1.

Allele frequencies of all SNPs were plotted for each isolate at every sample time point (Figs 3 and S1). Consistent with the summary shown by analysis of the *F*_WS_ indices, these plots show that in most isolate lines the genetic diversity gradually reduced over time in culture. Twenty-three parasite lines showed evidence of containing multiple genotypes in at least one of the early time points sampled. Of these, 13 had only a single genome detected by the end of the culture period, indicating that some *P. falciparum* genotypes outcompete others. This is apparent from the clearly parallel trajectory of allele frequency changes in most SNPs within the isolates, although the phase of haplotypes is not directly known by bulk sequencing. In some of the isolate lines, the frequency changes over time appear to follow a simple directional pattern (Fig. 3a). However, some of the lines displayed more complex changes in proportions of different genotypes over time (Fig. 3b). For example, the allele frequencies within line 284 indicate a minority genotype at day 25 that increased to majority at day 77, but then decreased to near disappearance by day 153.

### Drug resistance alleles do not explain most changes in genotype frequencies in culture

**Fig. 4.**
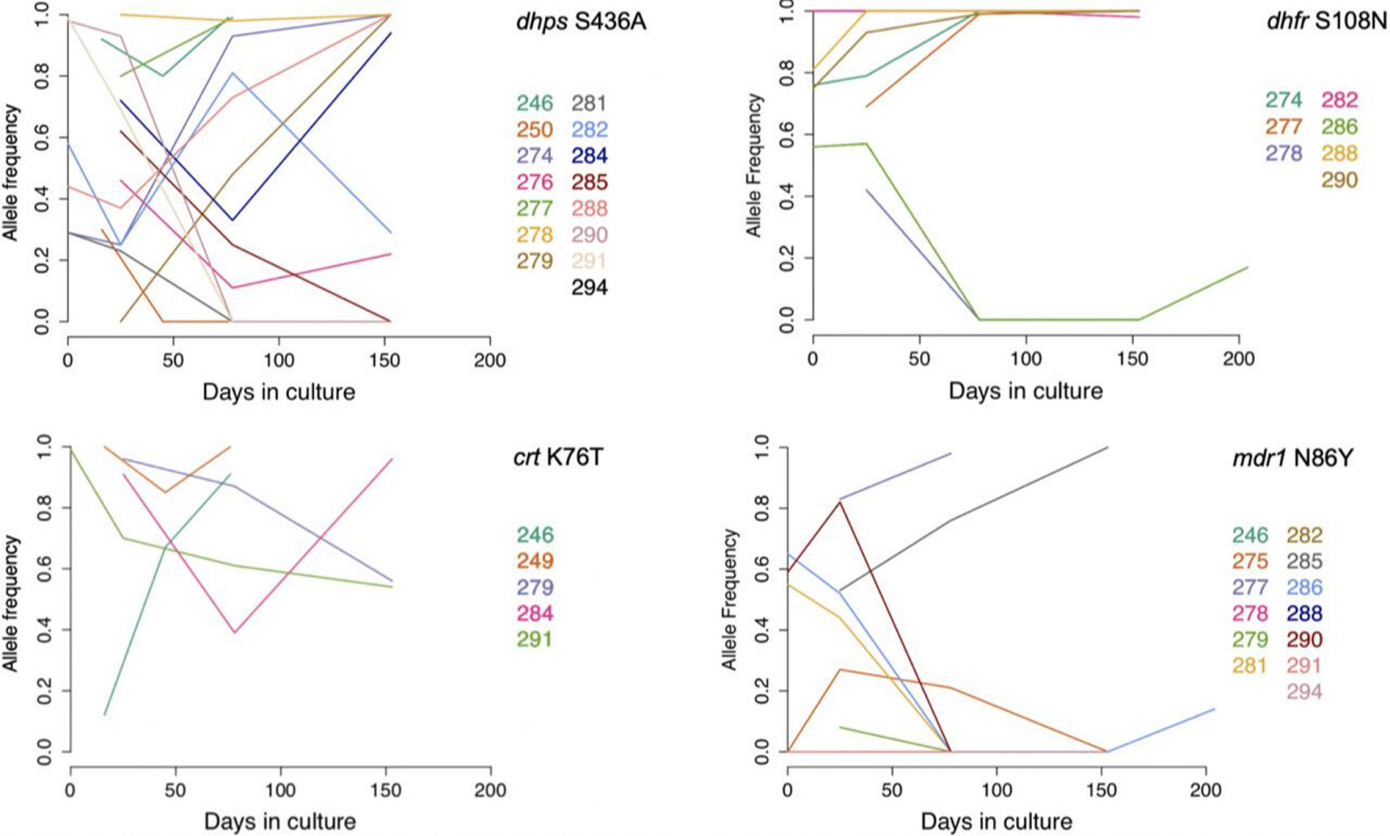
Frequencies of known drug resistance-associated alleles within *P. falciparum* multiple-genotype clinical isolates during several months of culture. Plots show data for one common resistance-associated codon polymorphism within each of four different genes (chloroquine resistance genes *crt* and *mdr1* on chromosomes 7 and 5, and antifolate resistance genes *dhfr* and *dhps* on chromosomes 4 and 8, respectively), with frequencies being estimated by relative proportions of read counts with each allele. For each of these polymorphisms, coloured lines indicate the data for isolates that had mixed alleles at one or more time points, each isolate labelled by its number in colour. The allele frequencies of other polymorphisms within these genes are shown in Fig. S2. Allele frequencies at each time point are shown fully in Table S2. Across all isolates, there was no significant directionality to the changes in frequencies of any of these resistance-associated polymorphisms over time in culture.

As drug resistance alleles may carry a fitness cost compared to wild-type alleles, we assessed their frequencies within polyclonal isolates during culture adaptation (Figs 4 and S2). A consistent decrease in resistant allele frequencies over time could indicate that genomes bearing wild-type alleles have a growth advantage. Four genes that have established drug resistance alleles circulating in Africa were examined (*crt*, *mdr1*, *dhfr* and *dhps*), with allele frequencies within cultures being estimated by the proportions of sequence reads corresponding to each allele. The main drug resistance-related SNP allele for each gene is shown in Fig. 4, and other variants in these genes are plotted in Fig. S2 (all allele frequencies for each time point of each isolate are given in Table S2). The *dhps* codon S436A is a marker of resistance to sulphadoxine, *dhfr* codon S108N resistance to pyrimethamine, *crt* K76T resistance to chloroquine and *mdr1* N86Y resistance to aminoquinolines including chloroquine. Out of 13 isolates mixed for the *dhps* S436A polymorphism, 7 showed a decrease and 6 an increase in the resistance-associated allele frequency during culture adaptation. For the *dhfr* S108N polymorphism, out of six mixed isolates two showed a decrease and four an increase in the resistance-associated allele frequency. Similarly, no *mdr1* or *crt* drug-resistant allele showed a trend in allele frequency change across the different isolates. Over all four genes, there was no evidence of a significant decrease in drug resistance marker allele frequencies over time, and no overall directionality to allele frequency changes (binomial test, *P*>0.5).

### Loss-of-function mutations emerging during culture

**Fig. 5.**
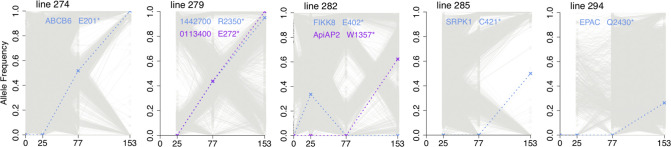
Emergence of premature stop codon mutants during culture of mixed-genotype *P. falciparum* clinical isolates. Stop codon alleles that emerged and attained a frequency of at least 0.2 are shown, with dotted lines and labelling of the gene and codon affected. Grey lines indicate all other SNP allele frequencies changing over time within these isolates. Separate to these new findings from mixed-genotype isolates, emerging mutants in three of the single-genotype isolates (lines 271, 272 and 280) were previously reported [4]. Apart from these, no other isolate among the 28 studied here had a loss-of-function mutant sequence detected to reach a frequency of 0.2 during culture.

In previous studies on single-genotype infection isolates, loss-of-function mutants appeared to have a selective advantage during culture growth. Here, to examine mixed-genotype isolates, analysis was conducted on stop codon mutations and indels generating frameshifts, as either type of mutation would lead to loss of function. Eight isolates showed at least one loss-of-function mutant rising in frequency during culture. Three of these were single-genotype isolates for which we had previously described the mutants [4] and five of them were multiple-genotype isolates for which the mutants were not previously described (Fig. 5 and Table 1).

**Table 1.** Novel mutants encoding premature stop codons emerging in multiple-clone *P. falciparum* clinical isolates

				Frequency at different culture time points			
**Isolate**	**Gene ID**	**Gene Name and Annotation**	**Codon**	**Day 0**	**Day 25**	**Day 77**	**Day 153**
**274**	PF3D7_1352100	ABCB6, ABC transporter B family member 6	E201*	0	0.00	0.52	1.00
**279**	PF3D7_1442700	Anonymous, conserved plasmodium protein	R2350*	*na*	0.00	0.43	0.95
**279**	PF3D7_0113400	Anonymous, plasmodium exported protein	E272*	*na*	0.00	0.44	1.00
**282**	PF3D7_0805700	FIKK8, serine/threonine protein kinase	E402*	0	0.34	0.00	0.00
**282**	PF3D7_1342900	AP2-HS, AP2 domain transcription factor	W1357*	0	0.00	0.00	0.62
**285**	PF3D7_0302100	SRPK1, serine/threonine protein kinase	C421*	*na*	0.00	0.00	0.50
**294**	PF3D7_1417400	EPAC, cyclic nucleotide-binding protein	Q2430*	0	0.00	0.00	0.26

Each of these mutants may be considered separately. The genes affected in two of the isolates have not been previously seen to have loss-of-function mutants emerge in culture. In isolate line 274, a premature stop codon in an ABC transporter gene *ACCB6* (at codon position 201) was associated with the genome sequence of an initial minority parasite that was undetectable at day 25, which replaced parasites with other genomes to become fixed within the culture by day 153. No stop codon is seen in this gene in sequences from several thousand clinical *P. falciparum* infections [6], and the orthologous gene is essential for *in vivo* replication of the rodent malaria parasite *Plasmodium berghei* in mice [18]. Interestingly, an insertional mutagenesis screen of the laboratory-adapted *P. falciparum* strain NF54 indicated that disruption of this gene strongly reduced the parasite growth rate in culture [19], which suggests that fitness effects are conditional on the parasite genetic background. In isolate line 279, there was a replacement of parasites with different genomes over time between days 25 and 153, with stop codon mutations in two different genes being associated with the genotype going to fixation. One of these genes (Pf3D7_0113400) encodes an exported protein [20], while the other encodes a protein of unknown function (Table 1). Insertional mutagenesis has previously indicated that both genes are dispensable for the growth of parasites in culture [19]. In one isolate a premature stop codon in a serine/threonine kinase gene *FIKK8* was seen in a minority of sequence reads at day 25 of culture, but not detected in any of the earlier or later time points, an observation not replicated or associated with directional change (Table 1).

In isolate lines 282, 285 and 294, premature stop codons were detected respectively in *ApiAP2-HS*, *SRPK1* and *EPAC* (Fig. 5 and Table 1). In each case, the mutants were not seen until after day 77 of culture and had intermediate frequencies that were still far from fixation by day 153. Other independent premature stop codon mutations were previously seen to emerge in these same three genes during culture of single-genotype Gambian clinical isolates [3], and two of these genes (*ApiAP2-HS* and *EPAC*) had premature stop codon mutations emerging during culture of the single-genotype Ghanaian isolates [4]. It is likely that a slight growth fitness advantage is conferred by loss-of-function mutation of these genes, as mutants have emerged on different single-genome isolate backgrounds, where they were not associated with other genomic changes, as well as in the mixed-genotype isolates here.

As no loss-of-function mutants were detected during culture of the other uncloned clinical isolates analysed here, the overall proportion of isolates with such emerging mutants was 29 % (8 out of 28). There was no significant difference in the proportions for single-genotype isolates (38 %, 3 out of 8) and multiple-genotype isolates (25 %, 5 out of 20) (Fisher’s exact test, *P*=0.65).

### Identification of *de novo* mutations in parasite clones

**Fig. 6.**
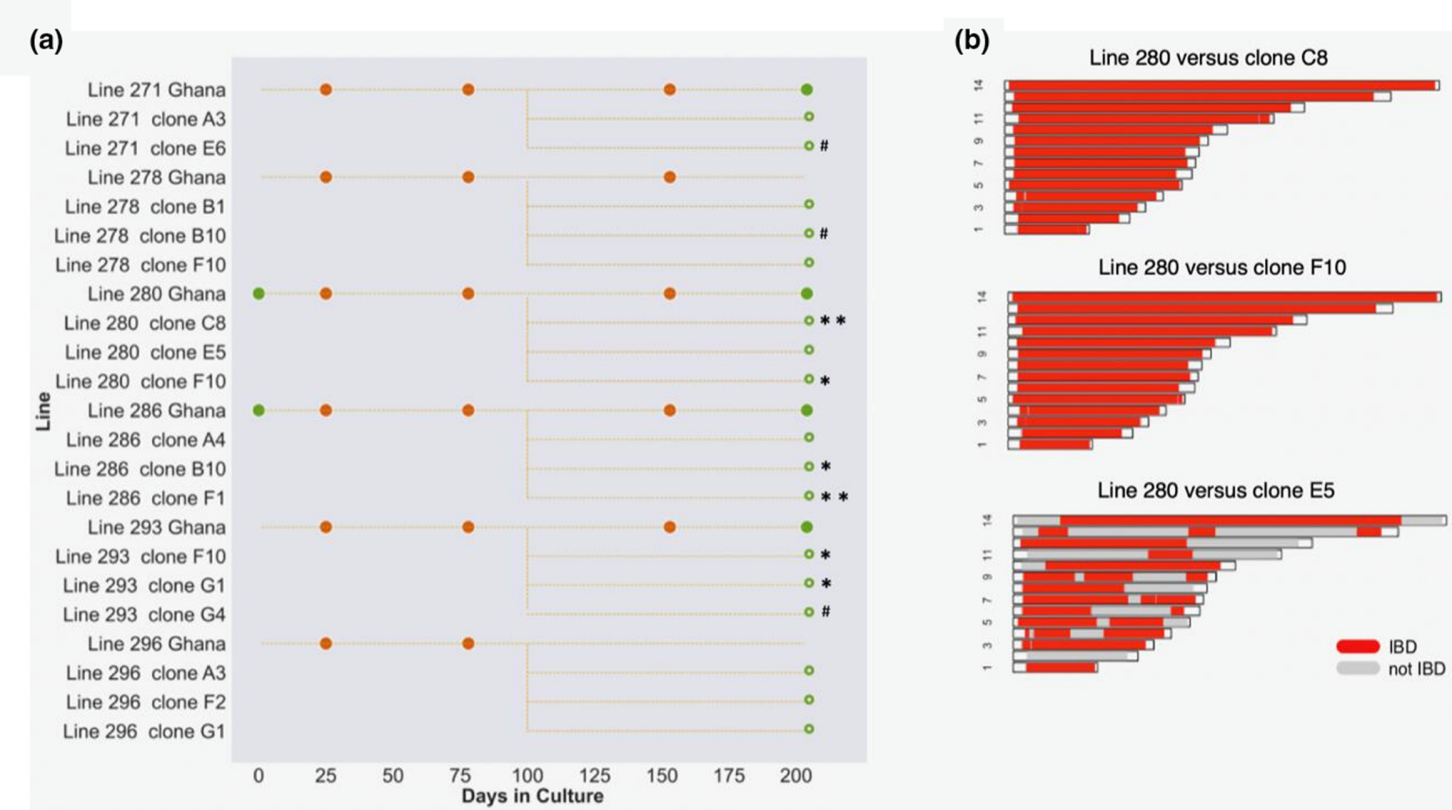
Genome sequencing of parasite clones derived from isolates show additional novel mutants as well as sibling parasites. (**a**) After 100 days of culture, clones were derived from 6 of the clinical isolate lines by limiting dilution, and 104 days after the cloning step a few of these clones from each isolate were sampled for sequencing (17 in total), indicated by hollow green circles. These sequences were compared with the bulk sequence of the corresponding isolate (at day 77). Clones in which novel SNPs and indels were detected are indicated by asterisks and hashtags respectively (the mutants are detailed in Table 2). (**b**) Clones from each isolate were scanned for genomic patterns of identity by descent (IBD). The 3 panels show the IBD in the 14 chromosomes, comparing the bulk-cultured line 280 (at day 77) with 3 derived clones. Sub-telomeric regions at the end of each chromosome were not analysed and are unshaded. Clone E5 shows 60.8 % of the genome in IBD with the day 77 genome as well as with clones 1 and 2, consistent with a full sibling relationship. The clones from the other isolates showed complete identity to their respective isolate bulk culture majority sequences, although deeper sampling would likely reveal more cases of non-identical sibling parasites.

Bulk whole-genome sequencing of uncloned isolates may only be likely to detect new parasite variants under positive selection, as most new mutants will be initially very rare. As an alternative qualitative approach to investigate whether other variants may be present, we performed parasite cloning by limiting dilution of six of the clinical isolates after 100 days of culture. The parasite clones were then grown for up to 104 days, and for an initial scan up to 3 of these clones from each isolate were sequenced, generating 17 clone sequences in total (Fig. 6a and Table S3). Novel SNPs or indels were detected in 11 of the 17 clones (Table 2). Interestingly, four of these are loss-of-function mutations in the *EPAC* gene, a different novel variant being detected in a clone from each of four different isolates. This is further evidence that such mutants commonly arise within the *EPAC* gene, the locus with the most loss-of-function mutants previously detected. Among the other loci with a novel variant is gene locus Pf3D7_1320700 with a nonsynonymous change (Table 2), potentially notable as this same gene has a premature stop codon mutant in the long-term culture-adapted line Dd2 [3].

**Table 2.** Sequence variants identified in parasites cloned from clinical isolates

Isolate clone	Gene ID	Gene name and annotation	Variant and effect
**271 E6**	PF3D7_1417400	EPAC, rap guanine nucleotide exchange factor	Indel frameshift
**278 B10**	PF3D7_0217600	Unknown function	Indel in intron
**280** C8	PF3D7_0822400	Unknown function	Non-synonymous SNP D93E
**280** F10	PF3D7_1204100	Unknown function	Non-synonymous SNP R918G
**280** C8	PF3D7_1417400	EPAC, rap guanine nucleotide exchange factor	Premature stop codon E1422*
**286** F1	PF3D7_0415200	Unknown function	Non-synonymous SNP K2122T
**286** F1	PF3D7_0615500	CRK5, cdc2-related protein kinase 5	Non-synonymous SNP S101N
**286 B10**	PF3D7_1439100	DEAD/DEAH box helicase	Non-synonymous SNP K641T
**293** F10	PF3D7_1320700	Unknown function	Non-synonymous SNP Q1263R
**293** G4	PF3D7_1417400	EPAC, rap guanine nucleotide exchange factor	Indel frameshift
**296** G1	PF3D7_1417400	EPAC, rap guanine nucleotide exchange factor	Premature stop codon K3366*

### Identification of sibling parasites in clones of a clinical isolate

To determine the relatedness between clones generated from the same isolate, the level of genomic IBD was calculated [17], which considers the segments of chromosomes shared between haploid genomes. As expected, most clones from the same isolate had virtually identical genome sequences, reflecting clones of the same common genotype within an isolate. More interestingly, clone E5 from isolate line 289 showed 60.8 % IBD with the other two clones that were derived at the same time (Fig. 6b), and with the majority genome in the bulk culture of the isolate at days 78 and 153. Based on allele frequencies in the bulk isolate sequence (Fig. S1), the proportion of clone 3 was approximately 5 % at day 78 of culture, prior to cloning. This clone is a sibling of the majority genome, with 25 recombination breakpoints observed between them, similar to the average number among progeny from an experimental cross [21]. This proportion of genetic relatedness among parasites from the same isolate is within the range that was previously estimated by single-cell sequencing [7] or by multi-locus genomic analysis of cloned parasites from clinical infections elsewhere in Africa [22].

## Discussion

These results, which focus mostly on multiple-genotype clinical isolates, extend previous findings from studies of single-genotype isolates, showing that loss-of-function mutants appear over time in culture. Overall, 8 (29 %) of the 28 isolates analysed had a premature stop codon or frameshift mutant arising and significantly increasing in frequency during several months of culture. All these isolates were cultured in the same laboratory, and there was no significant difference between single-genotype and multiple-genotype isolates in the proportions affected. The proportion affected was also not significantly different from that previously seen in a separate analysis of single-genotype isolates cultured in a different laboratory, in The Gambia [3]. The variants were first detected after different lengths of time in culture, but only very rarely within the first 2 months. If future studies on clinical isolates are to avoid the potential of phenotypes being affected by mutants emerging in culture, it may be preferable to focus on analysing parasites that have not been in culture for longer than approximately this length of time. Many isolates that have been cultured for longer might still be mutant-free, but genome sequencing to screen for loss-of-function mutants may be important as a quality control if longer-term cultures are analysed.

Most of the multiple-genotype isolates showed overall reduction of genetic diversity over culture time, which is not explained by drug resistance allele frequencies or loss-of-function mutations. Aside from genomic differences between parasites, it is likely that growth rates are modified by changes at an epigenetic level, and these may occur at different time points in different lineages. Previous analysis of exponential parasite multiplication rates in some of the isolates analysed here showed that the rates usually increased over time in culture, and that this was not associated with whether isolates had single or multiple genotypes [4].

From previous analyses of single-genotype clinical isolates, we identified 9 *de novo* SNPs emerging and attaining within-isolate allele frequencies of more than 20 % [3, 4]. Seven of these SNPs encoded premature stop codons, mainly in the *EPAC* gene and in the *ApiAP2* gene family. Loss-of-function mutations in *EPAC* have previously been shown in long-term laboratory-adapted parasite lines [3], and experimental analysis has confirmed that the gene is not functionally involved in cyclic AMP signalling as had previously been considered [23]. Although such mutants have been repeatedly detected to emerge during culture of clinical isolates, it is interesting that the majority of isolates still do not carry such mutants even after up to 7 months in culture. The *P. falciparum* mutation rate is high enough that every individual nucleotide is likely to be mutated in at least one parasite of the total population within a continuous culture flask [1, 2], so it remains unclear why mutants are detected as emergent in some isolates and not others. There are two non-mutually exclusive potential explanations. Firstly, any individual novel variant that confers a small survival advantage is likely to be randomly lost from a population shortly after arising [24]. Indeed, most loss-of-function mutants identified here were first detected at day 153, and longer culture adaptation might lead to more cultured isolates having time to acquire a loss-of-function mutation in *EPAC*, as seen in many long-term laboratory-adapted strains [3]. Secondly, it is possible that such mutations in *EPAC* would only confer a fitness advantage in the appropriate genome background, in epistasis with other unknown genes, in which case some lines would never acquire a loss-of-function mutation in *EPAC*. It should be noted that bulk sequencing of multiple-genotype isolates is not usually sufficient for identifying whether loss-of-function mutants are under selection, as it may not be possible to separate the effect of such a variant from other genomic differences.

A pertinent question is whether particular loss-of-function gene mutants would appear in biological replicate cultures of any given isolate. Investigating patterns of sequence changes in replicate cultures of each isolate would be a potential approach for future prospective study. As an extension of this, controlled changes in culture adaptation conditions, such as temperature or composition of culture media, could be explored to test whether any particular type of mutant emergence is associated with specific conditions. The amount of continuous culture handling time and effort required for each isolate of this parasite has precluded taking such an approach yet, and future studies involving independent replicates would probably need to focus on fewer isolates, the scale of which may be guided by information reported here.

Acquiring drug resistance may be at the expense of fitness in an environment where the drug is absent, but the relationship between resistance alleles and fitness is not straightforward, particularly as isolates that contain resistance alleles may also have inherited compensatory mutations elsewhere in the genome. Evidence that drug resistance has a fitness cost is illustrated by chloroquine resistance in Africa, where chloroquine use declined after resistance reached high frequency, following which the *crt* K76T resistance allele frequency declined in many populations, indicating a fitness cost in the absence of drug selection [25]. *In vitro*, fitness costs may be detected by differential growth rates, which is subject to experimental conditions and also depends on the genomic backgrounds of the parasites, as illustrated by independent studies on variants of the *mdr1* gene [26, 27]. It is becoming increasingly clear that epistasis between variants of different genes contribute to parasite fitness, particularly in the context of drug resistance selection and associated fitness costs [28, 29], but also due to selective processes that are less well known [29].

Within a culture of any multiple genotype infection isolate, all parasite genotypes are subject to the same conditions, making this an appropriate setting for studying the genetic basis of growth competition. As illustrated here, faster growing lines tend to outcompete others, but the interaction is not necessarily linear. Generating data with larger sample sizes may enable future genome-wide association studies, by comparing slow growing genomes versus faster growing genomes and identifying allele frequencies significantly associated with the phenotype. For example, adjusting culture parameters such as temperature [5], static or shaking conditions [30], erythrocyte blood group types [31], or nutrient concentrations [32], may be performed in future to test whether there may be selection for parasite variants suited to particular conditions. Further, as illustrated by the demonstration of genetically related clones within one of the isolate lines analysed here and also within isolates studied elsewhere [22], analysing large numbers of related clones from within infections is a potential future approach to help associate genetic loci with phenotypes, in a similar manner to a quantitative trait loci analysis from genetic crosses [33].

## Supplementary Data

Supplementary material 1
