# Supplementary material for: Genomic variation during culture adaptation of genetically complex Plasmodium falciparum clinical isolates
Source: Microb Genom. 2023 May 19;9(5):mgen001009. doi: 10.1099/mgen.0.001009 (PMC10272863; doi:10.1099/mgen.0.001009)

Supplementary Information File

Contains three Supplementary Tables and two Supplementary Figures

Genomic variation during culture-adaptation of genetically complex *Plasmodium falciparum* clinical isolates

Antoine Claessens[1,2,3*], Lindsay B. Stewart[2], Eleanor Drury[4], Ambroise D. Ahouidi[5], Alfred Amambua-Ngwa[3], Mahamadou Diakite[6], Dominic P. Kwiatkowski[4], Gordon A. Awandare[7] & David J. Conway[2*]

Supplementary Table S1. Sources, accession numbers, genome-wide SNP coverage, and F_{WS} within-isolate fixation index values from Illumina short-read sequences of cultured clinical isolates at each timepoint (days in culture ranging from 0 to 204).

Country	Patient isolate	Days in culture	Data source	SangerID	ENA accession number	Coverage (%)	Fws fixation index
Sénégal	231	18	This study	SPT18853	ERR2496523	53.84	0.989
Sénégal	231	67	This study	SPT18854	ERR2496524	64.02	0.986
Sénégal	231	100	This study	SPT18855	ERR2234992	86.49	0.985
Guinea	246	16	This study	SPT18844	ERR2496521	82.12	0.666
Guinea	246	45	This study	SPT18845	ERR2436117,ERR2436129,ERR2436141	91.36	0.705
Guinea	246	76	This study	SPT18846	ERR2234991	88.05	0.951
Guinea	249	16	This study	SPT18847	ERR2508999	91.05	0.98
Guinea	249	45	This study	SPT18848	ERR2509000	90.02	0.963
Guinea	249	76	This study	SPT18849	ERR2436118,ERR2436130,ERR2436142	90.53	0.983
Mali	250	16	This study	SPT18850	ERR2509001	91.55	0.793
Mali	250	45	This study	SPT18851	ERR2509004	91.11	0.976
Mali	250	76	This study	SPT18852	ERR2496522	84.3	0.984
Ghana	271	25	Previous	SPT18770	ERR2234984	86.72	0.987
Ghana	271	78	Previous	SPT18771	ERR2496504	53.91	0.992
Ghana	271	153	Previous	SPT18820	ERR2234997	88.66	0.987
Ghana	271	204	This study	SPT18856	ERR2496525	56.55	0.991
Ghana	272	0	This study	SPT18883	ERR2509011,ERS311772	71.3	0.838
Ghana	272	25	Previous	SPT18772	ERR2234970	89.41	0.975
Ghana	272	78	Previous	SPT18773	ERR2508979	91.09	0.985
Ghana	272	153	Previous	SPT18821	ERR2508991	91.09	0.972
Ghana	273	0	This study	SPT18882	ERR2234998	89.05	0.985
Ghana	273	25	Previous	SPT18774	ERR2234999	89.4	0.812
Ghana	273	78	Previous	SPT18775	ERR2508980	90.93	0.984
Ghana	273	153	Previous	SPT18822	ERR2496509	65.22	0.99
Ghana	274	0	This study	PF0720-C	ERS311778	83.56	0.281
Ghana	274	25	Previous	SPT18776	ERR2235001	89.33	0.304
Ghana	274	78	Previous	SPT18777	ERR2508981	91.85	0.472
Ghana	274	153	Previous	SPT18823	ERR2496510	52.63	0.995
Ghana	275	25	Previous	SPT18778	ERR2234972	88.67	0.762
Ghana	275	78	Previous	SPT18779	ERR2234974	86.89	0.829
Ghana	275	153	Previous	SPT18824	ERR2496511	63.05	0.99
Ghana	276	25	Previous	SPT18780	ERR2436108,ERR2436120,ERR2436132	91.34	0.748
Ghana	276	78	Previous	SPT18781	ERR2508982	91.41	0.88
Ghana	276	153	Previous	SPT18825	ERR2496512	61.14	0.857
Ghana	277	25	Previous	SPT18782	ERR2508983	91.13	0.617
Ghana	277	78	Previous	SPT18783	ERR2234976	87.44	0.976
Ghana	278	25	Previous	SPT18784	ERR2235003	88.99	0.534
Ghana	278	78	Previous	SPT18785	ERR2508984	90.99	0.975
Ghana	278	153	Previous	SPT18826	ERR2234986	89.63	0.99
Ghana	279	25	Previous	SPT18786	ERR2234978	88.87	0.837
Ghana	279	78	Previous	SPT18787	ERR2235005	88.03	0.544

Ghana	279	153	Previous	SPT18827	ERR2508992	89.9	0.916
Ghana	280	0	This study	SPT18886	ERR2509012,ERS311771	70.06	0.883
Ghana	280	25	Previous	SPT18788	ERR2234980	87.98	0.971
Ghana	280	78	Previous	SPT18789	ERR2234982	88.65	0.957
Ghana	280	153	Previous	SPT18828	ERR2234987	88.21	0.976
Ghana	280	204	This study	SPT18864	ERR2496529	61.85	0.982
Ghana	281	0	This study	SPT18887	ERR2509002	90.29	0.443
Ghana	281	25	Previous	SPT18790	ERR2234985	88.54	0.278
Ghana	281	78	Previous	SPT18791	ERR2234971	88.54	0.912
Ghana	282	0	This study	SPT18880	ERR2234994	89.7	0.62
Ghana	282	25	Previous	SPT18792	ERR2234973	88.29	0.364
Ghana	282	78	Previous	SPT18793	ERR2234975	88.42	0.789
Ghana	282	153	Previous	SPT18830	ERR2508993	91.33	0.661
Ghana	284	25	Previous	SPT18796	ERR2436109,ERR2436121,ERR2436133	90.68	0.727
Ghana	284	78	Previous	SPT18797	ERR2234977	86.97	0.516
Ghana	284	153	Previous	SPT18832	ERR2496515	68.71	0.957
Ghana	285	25	Previous	SPT18798	ERR2235006	89.64	0.382
Ghana	285	78	Previous	SPT18799	ERR2235007	89.86	0.607
Ghana	285	153	Previous	SPT18833	ERR2496516	52.58	0.984
Ghana	286	0	This study	PF0711-C	ERS311769	86.99	0.575
Ghana	286	25	Previous	SPT18800	ERR2508986	91.14	0.639
Ghana	286	78	Previous	SPT18801	ERR2436110,ERR2436122,ERR2436134	90.78	0.94
Ghana	286	153	Previous	SPT18834	ERR2234989	88.98	0.867
Ghana	286	204	This study	SPT18868	ERR2496533	63.28	0.763
Ghana	287	0	This study	PF0715-C	ERS311773	85.98	0.989
Ghana	287	25	Previous	SPT18802	ERR2235008	88.62	0.985
Ghana	287	78	Previous	SPT18803	ERR2234979	88.93	0.989
Ghana	287	153	Previous	SPT18835	ERR2508994	90.29	0.98
Ghana	288	0	This study	SPT18881	ERR2234996	90.26	0.289
Ghana	288	25	Previous	SPT18804	ERR2436111,ERR2436123,ERR2436135	91.32	0.293
Ghana	288	78	Previous	SPT18805	ERR2508987	90.68	0.71
Ghana	288	153	Previous	SPT18836	ERR2496517	65.28	0.99
Ghana	289	25	Previous	SPT18806	ERR2508988	91.02	0.985
Ghana	289	78	Previous	SPT18807	ERR2508989	90.92	0.979
Ghana	290	0	This study	SPT18884	ERR2234988	89.48	0.564
Ghana	290	25	Previous	SPT18808	ERR2436112,ERR2436124,ERR2436136	91.6	0.413
Ghana	290	78	Previous	SPT18809	ERR2234981	88.59	0.884
Ghana	290	153	Previous	SPT18838	ERR2496519	57.55	0.987
Ghana	291	0	This study	SPT18890	ERR2234995	89.54	0.979
Ghana	291	25	Previous	SPT18810	ERR2235009	89.84	0.477
Ghana	291	78	Previous	SPT18811	ERR2235000	88.92	0.978
Ghana	291	153	Previous	SPT18839	ERR2508995	90.01	0.951
Ghana	292	0	This study	PF0691-C	ERS311749	87.97	0.992
Ghana	292	25	Previous	SPT18812	ERR2234983	88.25	0.986
Ghana	292	78	Previous	SPT18813	ERR2496506	51.14	0.994
Ghana	292	153	Previous	SPT18840	ERR2496520	62.75	0.99
Ghana	293	25	Previous	SPT18814	ERR2235002	89.21	0.961
Ghana	293	78	Previous	SPT18815	ERR2235004	89.35	0.989
Ghana	293	153	Previous	SPT18841	ERR2508996	90.87	0.973
Ghana	293	204	This study	SPT18872	ERR2496535	66.43	0.989
Ghana	294	25	This study	SPT18816	ERR2508990	65.46	NA
Ghana	294	78	Previous	SPT18817	ERR2496507	NA	0.237
Ghana	294	153	Previous	SPT18842	ERR2508997	91.99	0.338
Ghana	296	25	Previous	SPT18818	ERR2436114,ERR2436126,ERR2436138	90.72	0.991
Ghana	296	78	Previous	SPT18819	ERR2496508	53.35	0.991

Reference for source of previous data: Stewart *et al.* 2020 *Communications Biology* 3:624

The Coverage (%) for each sample specifies the percentage of all SNPs in the core genome with a mapped sequence read depth of more than 5, among all SNPs called by the MalariaGEN pipeline. The F_{WS} fixation index here is based on analysis of SNPs with a read depth of at least 20 in each sample, which is a stringent minimum (similar trends across samples are seen if lower cut-off read depths of either 10 or 5 are applied). Overall, there is a negative correlation between SNP coverage and F_{WS} value (Pearson's r = -0.32) as some samples with relatively low coverage of <80% have lower power to detect within-isolate diversity, but this does not significantly affect the main analyses in this paper, as the level of coverage is not correlated with particular timepoints.

Supplementary Table S2. Drug resistance allele frequencies (in *dhfr*, *mdr1*, *crt* and *dhps* genes) within each cultured *P. falciparum* clinical isolate at each sampled timepoint, estimated by relative proportions of read counts with each alternative allele.

**Gene**	**DHFR**	**DHFR**	**DHFR**	**MDR1**	**MDR1**	**MDR1**	**CRT**	**DHPS**	**DHPS**	**DHPS**	**DHPS**
**CHR**	4	4	4	5	5	5	7	8	8	8	8
**POS**	748239	748262	748410	958145	958440	961625	403625	549681	549685	549993	550212
**REF**	A	T	G	A	A	G	A	G	T	A	G
**ALT**	T	C	A	T	T	T	C	C	G	G	T
**Mutant**	**N51I**	**C59R**	**S108N**	**N86Y**	**Y184F**	**D1246Y**	**K76T**	**S436A**	**G437A**	**K540E**	**A613S**
**Isolate & timepoint:**											
**231_d018**	NA	1.00	1.00	0.00	0.00	0.00	NA	0.00	0.00	0.00	0.00
**231_d067**	1.00	1.00	1.00	0.00	0.00	0.00	NA	0.00	0.00	0.00	NA
**231_d100**	1.00	1.00	1.00	0.00	0.00	0.00	0.00	0.00	0.00	0.00	NA
**246_d016**	1.00	1.00	1.00	0.00	0.97	0.00	0.12	0.92	0.92	0.00	0.00
**246_d045**	1.00	1.00	1.00	0.00	0.85	NA	0.67	0.80	0.80	0.00	0.00
**246_d076**	1.00	1.00	1.00	0.00	1.00	NA	0.91	0.99	0.99	0.00	0.00
**249_d016**	0.00	0.00	0.00	0.00	1.00	NA	1.00	0.00	0.00	0.00	NA
**249_d045**	NA	0.00	NA	0.00	1.00	0.00	0.85	NA	0.00	0.00	0.00
**249_d076**	0.00	0.00	0.00	0.00	1.00	NA	1.00	0.00	0.00	0.00	NA
**250_d016**	1.00	1.00	1.00	0.00	0.00	0.00	0.00	0.30	0.93	0.00	0.00
**250_d045**	1.00	1.00	1.00	0.00	0.00	0.00	0.00	0.00	1.00	0.00	0.00
**250_d076**	1.00	1.00	1.00	0.00	0.00	0.00	0.00	0.00	1.00	0.00	0.00
**271_d025**	1.00	1.00	1.00	0.00	0.99	NA	0.00	0.00	0.00	0.00	NA
**271_d078**	1.00	1.00	1.00	0.00	1.00	0.00	NA	0.00	0.00	0.00	0.00
**271_d153**	1.00	1.00	1.00	0.00	0.99	NA	0.00	0.00	0.00	0.00	NA
**271_d204**	1.00	1.00	1.00	0.00	1.00	0.00	NA	0.00	0.00	0.00	NA
**272_d000**	0.00	0.00	0.00	NA	NA	NA	NA	NA	NA	NA	NA
**272_d025**	0.00	0.00	0.00	0.00	0.00	NA	1.00	0.00	0.00	0.00	NA
**272_d078**	0.00	0.00	0.00	0.00	0.00	NA	1.00	0.00	0.00	0.00	NA
**272_d153**	0.00	0.00	0.00	0.00	0.00	NA	1.00	0.00	0.00	0.00	NA
**273_d000**	1.00	1.00	1.00	1.00	0.99	0.00	0.00	0.00	0.00	0.00	NA
**273_d025**	0.98	0.99	0.99	0.92	0.99	0.00	0.00	0.00	0.00	0.00	NA
**273_d078**	1.00	1.00	1.00	0.99	1.00	0.00	0.00	0.00	0.00	0.00	NA
**273_d153**	1.00	1.00	1.00	1.00	1.00	0.00	NA	0.00	0.00	0.00	NA
**274_d000**	0.73	0.71	0.76	0.00	NA	0.00	0.00	0.29	0.04	0.00	0.00
**274_d025**	0.81	0.81	0.79	0.11	0.98	0.09	0.00	0.25	0.00	0.00	0.00
**274_d078**	0.99	0.99	1.00	0.00	1.00	0.00	0.00	0.93	0.40	0.00	0.47
**274_d153**	NA	NA	1.00	NA	NA	0.00	NA	1.00	0.00	0.00	1.00
**275_d000**	1.00	1.00	1.00	0.00	0.00	0.00	0.00	0.00	0.00	0.00	0.00
**275_d025**	1.00	1.00	1.00	0.27	0.00	0.31	0.00	0.00	0.00	0.00	0.00
**275_d078**	1.00	1.00	1.00	0.21	0.00	0.35	0.00	0.00	0.00	0.00	0.00
**275_d153**	1.00	1.00	1.00	0.00	0.00	0.00	NA	0.00	0.00	0.00	0.00
**276_d025**	0.98	0.98	0.97	0.00	0.00	0.00	0.00	0.46	0.00	0.53	0.00
**276_d078**	1.00	1.00	0.99	0.00	0.00	0.00	0.00	0.11	0.00	0.88	0.00
**276_d153**	1.00	1.00	1.00	0.00	0.00	0.00	NA	0.22	0.00	0.68	0.00
**277_d025**	0.69	0.70	0.69	0.83	0.16	0.87	0.00	0.80	0.00	0.00	0.80
**277_d078**	1.00	1.00	0.99	0.98	0.00	1.00	0.00	0.99	0.00	0.00	0.97
**278_d025**	0.46	0.45	0.42	0.00	0.56	0.69	0.00	1.00	0.39	0.00	0.00
**278_d078**	0.00	0.00	0.00	0.00	1.00	1.00	0.00	0.98	0.00	0.00	0.00
**278_d153**	0.00	0.00	0.00	0.00	1.00	0.98	0.00	1.00	0.00	0.00	0.00
**279_d025**	0.95	1.00	0.99	0.08	0.98	0.00	0.96	0.00	0.13	0.00	0.12
**279_d078**	1.00	1.00	1.00	0.00	0.59	0.00	0.87	0.48	0.00	0.00	0.00
**279_d153**	1.00	1.00	1.00	0.00	0.00	NA	0.56	1.00	0.00	0.00	0.00
**280_d000**	NA	NA	0.94	NA	NA	NA	0.00	0.99	0.00	0.00	0.00
**280_d025**	1.00	1.00	1.00	0.00	0.99	0.00	0.00	0.99	0.00	0.00	0.00

**280_d078**	1.00	1.00	1.00	0.00	0.99	0.00	0.00	0.99	0.00	0.00	0.00
**280_d153**	0.99	0.99	1.00	0.00	1.00	0.00	0.00	1.00	0.00	0.00	0.00
**280_d204**	1.00	1.00	1.00	0.00	1.00	0.00	NA	1.00	0.00	0.00	0.00
**281_d000**	0.98	0.99	0.97	0.55	0.95	0.00	0.00	0.29	0.00	0.00	0.28
**281_d025**	NA	0.95	0.94	0.44	0.62	0.00	0.00	0.23	NA	0.00	0.14
**281_d078**	0.95	0.94	0.97	0.00	0.00	0.00	0.00	0.00	0.00	0.00	NA
**282_d000**	0.61	1.00	1.00	0.00	0.99	0.00	0.00	0.58	0.00	0.00	0.00
**282_d025**	0.64	1.00	1.00	0.00	0.82	0.00	0.00	0.25	0.17	0.00	0.22
**282_d078**	0.88	1.00	1.00	0.00	1.00	0.00	0.00	0.81	0.00	0.00	0.00
**282_d153**	0.14	1.00	0.98	0.00	0.96	0.00	0.00	0.29	0.00	0.00	0.00
**284_d025**	0.00	1.00	1.00	0.00	0.93	0.00	0.91	0.72	0.00	0.00	0.71
**284_d078**	NA	0.98	0.99	0.00	0.99	0.00	0.39	0.33	0.00	0.00	0.31
**284_d153**	0.00	1.00	1.00	0.00	1.00	0.00	0.96	0.94	0.00	0.00	0.99
**285_d025**	1.00	1.00	1.00	0.53	0.63	0.00	0.00	0.62	0.00	0.00	0.00
**285_d078**	1.00	1.00	1.00	0.76	0.70	0.00	0.00	0.25	0.00	0.00	0.00
**285_d153**	1.00	1.00	1.00	1.00	1.00	0.00	NA	0.00	0.00	0.00	0.00
**286_d000**	0.62	0.63	0.56	0.65	1.00	0.00	0.00	NA	0.00	0.00	0.00
**286_d025**	0.49	0.46	0.57	0.52	1.00	0.00	0.00	0.00	0.00	0.00	0.00
**286_d078**	0.00	0.00	0.00	0.00	1.00	NA	0.00	0.00	0.00	0.00	0.00
**286_d153**	0.00	0.00	0.00	0.00	1.00	NA	0.00	0.00	0.00	0.00	0.00
**286_d204**	0.11	0.10	0.17	0.14	1.00	0.00	NA	0.00	0.00	0.00	0.00
**287_d000**	1.00	1.00	1.00	1.00	1.00	0.97	0.00	0.00	NA	0.00	0.00
**287_d025**	1.00	1.00	1.00	1.00	1.00	1.00	0.00	0.00	0.00	0.00	0.00
**287_d078**	1.00	1.00	1.00	1.00	0.99	1.00	0.00	0.00	0.00	0.00	0.00
**287_d153**	1.00	1.00	1.00	0.81	NA	1.00	NA	NA	0.00	0.00	0.00
**288_d000**	0.78	0.78	0.81	0.00	0.39	NA	0.00	0.44	0.00	0.00	0.00
**288_d025**	1.00	1.00	1.00	0.00	0.73	NA	0.00	0.37	0.00	0.00	0.00
**288_d078**	0.98	0.98	1.00	NA	NA	0.00	0.00	0.73	0.00	0.00	0.00
**288_d153**	1.00	1.00	1.00	0.00	1.00	NA	NA	1.00	0.00	0.00	0.00
**289_d025**	1.00	1.00	0.99	0.00	1.00	0.00	0.98	0.00	0.00	0.00	0.00
**289_d078**	1.00	1.00	1.00	0.00	1.00	0.00	0.96	0.00	0.00	0.00	0.00
**290_d000**	0.09	0.75	0.75	0.59	0.36	0.68	0.00	0.98	0.18	0.00	0.00
**290_d025**	0.34	0.92	0.93	0.82	0.39	0.50	0.00	0.93	0.00	0.00	0.00
**290_d078**	0.90	0.98	0.99	0.00	0.00	0.00	0.00	0.00	0.00	0.00	0.00
**290_d153**	1.00	1.00	1.00	0.00	0.00	0.00	NA	0.00	0.00	0.00	0.00
**291_d000**	1.00	1.00	1.00	0.00	0.99	NA	0.99	0.99	0.00	0.00	0.97
**291_d025**	1.00	1.00	0.99	0.00	0.33	0.00	0.70	0.69	0.00	0.00	0.66
**291_d078**	1.00	1.00	1.00	0.00	0.00	0.00	0.61	0.00	0.00	0.00	0.00
**291_d153**	1.00	1.00	NA	0.00	0.00	0.00	0.54	NA	0.00	0.00	0.00
**292_d000**	1.00	1.00	1.00	1.00	1.00	0.00	0.00	1.00	0.00	0.00	0.00
**292_d025**	1.00	1.00	1.00	0.97	0.99	0.00	0.00	1.00	0.00	0.00	0.00
**292_d078**	NA	NA	1.00	NA	1.00	0.00	NA	1.00	0.00	0.00	0.00
**292_d153**	1.00	1.00	1.00	1.00	1.00	0.00	NA	1.00	0.00	0.00	0.00
**293_d025**	0.00	1.00	1.00	0.00	0.98	NA	0.00	0.00	0.00	0.00	0.00
**293_d078**	0.00	1.00	1.00	0.00	1.00	NA	0.00	0.00	0.00	0.00	0.00
**293_d153**	0.00	0.99	0.98	0.00	1.00	0.00	0.00	0.00	0.00	0.00	0.00
**293_d204**	0.00	1.00	1.00	0.00	1.00	NA	0.00	0.00	0.00	0.00	0.00
**294_d078**	0.99	1.00	1.00	0.00	0.94	0.00	0.00	0.24	0.37	0.00	0.20
**294_d153**	1.00	1.00	1.00	0.00	0.78	0.00	0.00	0.18	0.21	0.00	0.17
**296_d025**	1.00	1.00	1.00	0.00	1.00	NA	0.00	1.00	0.00	0.00	NA
**296_d078**	1.00	1.00	1.00	0.00	1.00	0.00	NA	1.00	0.00	0.00	0.00

Supplementary Table S3. Sources, accession numbers and read depth of genomes of all cloned isolates. Clones were generated after day 100 of clinical isolate cultures, and sampled for sequencing after another 104 days in culture.

SampleID	Data source	SangerID	ENA accession number	Coverage (%)
271_CloneA3	This study	SPT18859	ERR2496527	55.58
271_CloneE6	This study	SPT18857	ERR2496526	63.41
278_CloneB1	This study	SPT18863	ERR2509008	90.69
278_CloneB10	This study	SPT18861	ERR2509006	90.74
278_CloneF10	This study	SPT18862	ERR2509007	90.55
286_CloneA4	This study	SPT18869	ERR2509009	90.73
286_CloneB10	This study	SPT18871	ERR2496534	57.68
286_CloneF1	This study	SPT18870	ERR2509010	90.96
280_CloneC8	This study	SPT18865	ERR2496530	56.62
280_CloneE5	This study	SPT18866	ERR2496531	54.41
280_CloneF10	This study	SPT18867	ERR2496532	60.43
293_CloneF10	This study	SPT18874	ERR2496537	64.05
293_CloneG1	This study	SPT18873	ERR2496536	63.86
293_CloneG4	This study	SPT18875	ERR2496538	70.87
296_CloneA3	This study	SPT18877	ERR2496540	52.09
296_CloneF2	This study	SPT18879	ERR2496542	62.89
296_CloneG1	This study	SPT18878	ERR2496541	58.43

Supplementary Fig. S1. Genome-wide plots of single nucleotide polymorphism allele frequencies within *P. falciparum* clinical isolates during culture adaptation. All isolates are shown, with plots as described in the legend of Fig. 3.

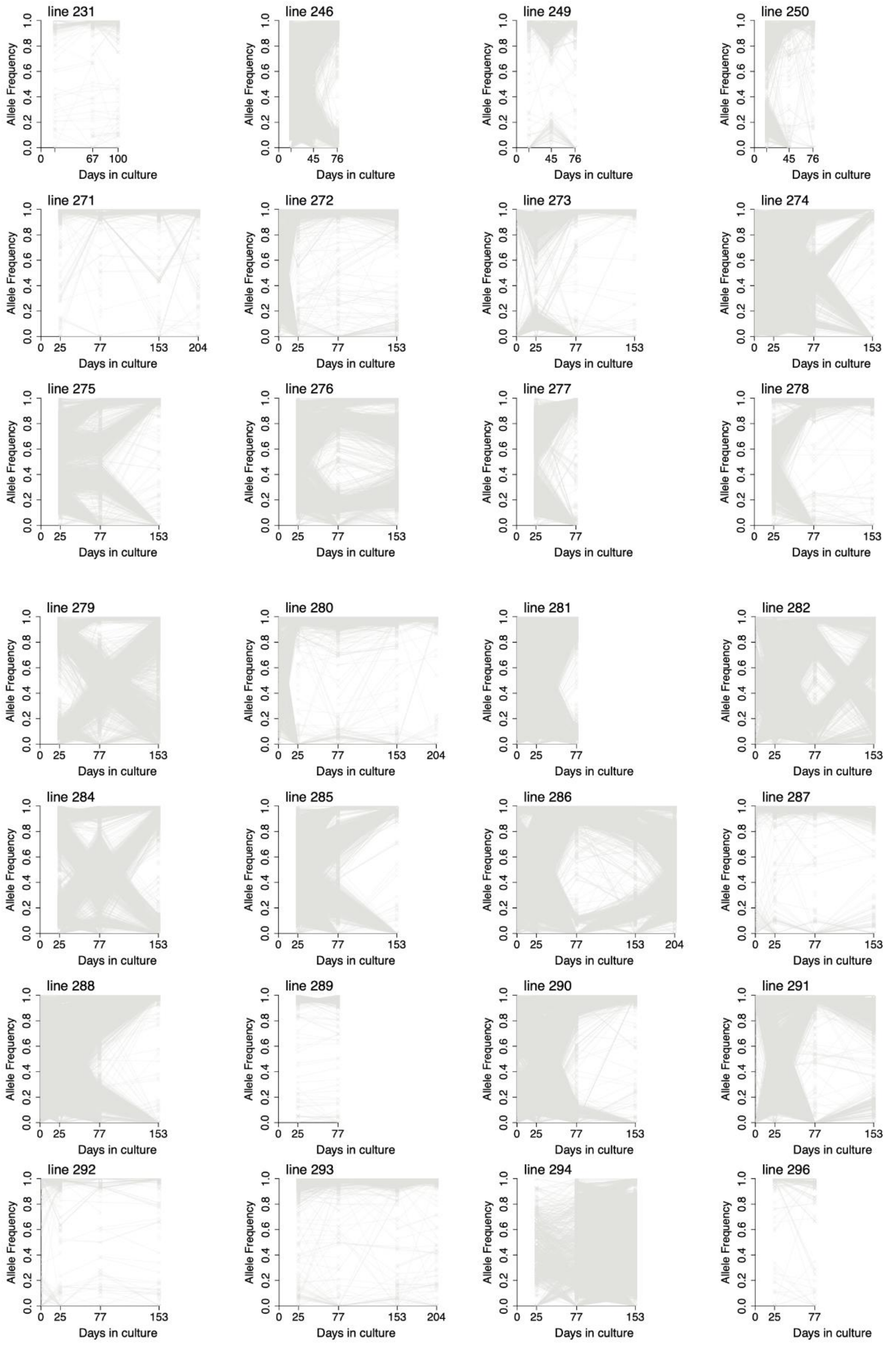

Supplementary Fig. S2. Allele frequencies at SNPs in *P. falciparum* drug resistance genes *dhfr*, *dhps* and *mdr1*, additional to those shown in Fig. 4, in multiple-clone isolates after different lengths of time in culture. For each polymorphism, isolates shown are only those that had mixed alleles at one or more timepoints. Across all isolates, there was no significant directionality to the changes in frequencies of any of these resistance-associated polymorphisms over time in culture. Allele frequencies at each timepoint are shown in Supplementary Table S2.

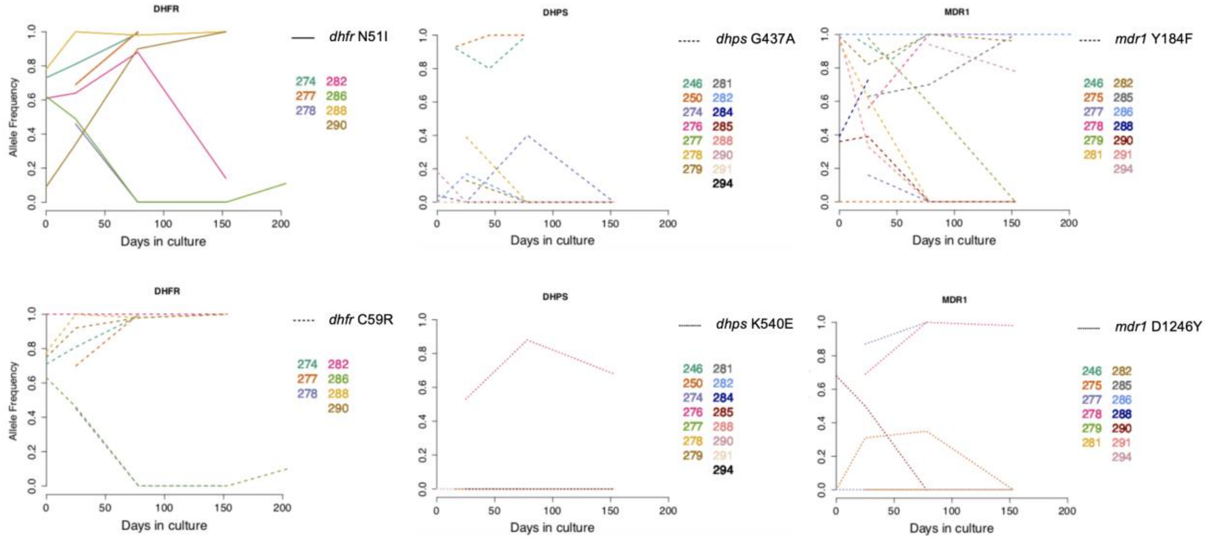

Supplement: Supplementary material 1 [file mgen-9-1009-s001.pdf]
